# Extending outbreak investigation with machine learning and graph theory: Benefits of new tools with application to a nosocomial outbreak of a multidrug-resistant organism

**DOI:** 10.1017/ice.2022.66

**Published:** 2023-02

**Authors:** Andrew Atkinson, Benjamin Ellenberger, Vanja Piezzi, Tanja Kaspar, Luisa Salazar-Vizcaya, Olga Endrich, Alexander B. Leichtle, Jonas Marschall

**Affiliations:** 1 Department of Infectious Diseases, Bern University Hospital, Inselspital, University of Bern, Bern, Switzerland; 2 Insel Data Science Center, Bern University Hospital, Inselspital, University of Bern, Bern, Switzerland; 3 Medical Directorate, Bern University Hospital, Inselspital, University of Bern, Bern, Switzerland; 4 University Institute of Clinical Chemistry, Bern University Hospital, Inselspital, University of Bern, Bern, Switzerland; 5 Division of Infectious Diseases, Washington University School of Medicine, St Louis, Missouri, United States

## Abstract

**Objective:**

From January 1, 2018, until July 31, 2020, our hospital network experienced an outbreak of vancomycin-resistant enterococci (VRE). The goal of our study was to improve existing processes by applying machine-learning and graph-theoretical methods to a nosocomial outbreak investigation.

**Methods:**

We assembled medical records generated during the first 2 years of the outbreak period (January 2018 through December 2019). We identified risk factors for VRE colonization using standard statistical methods, and we extended these with a decision-tree machine-learning approach. We then elicited possible transmission pathways by detecting commonalities between VRE cases using a graph theoretical network analysis approach.

**Results:**

We compared 560 VRE patients to 86,684 controls. Logistic models revealed predictors of VRE colonization as age (aOR, 1.4 (per 10 years), with 95% confidence interval [CI], 1.3–1.5; *P* < .001), ICU admission during stay (aOR, 1.5; 95% CI, 1.2–1.9; *P* < .001), Charlson comorbidity score (aOR, 1.1; 95% CI, 1.1–1.2; *P* < .001), the number of different prescribed antibiotics (aOR, 1.6; 95% CI, 1.5–1.7; *P* < .001), and the number of rooms the patient stayed in during their hospitalization(s) (aOR, 1.1; 95% CI, 1.1–1.2; *P* < .001). The decision-tree machine-learning method confirmed these findings. Graph network analysis established 3 main pathways by which the VRE cases were connected: healthcare personnel, medical devices, and patient rooms.

**Conclusions:**

We identified risk factors for being a VRE carrier, along with 3 important links with VRE (healthcare personnel, medical devices, patient rooms). Data science is likely to provide a better understanding of outbreaks, but interpretations require data maturity, and potential confounding factors must be considered.

Electronic medical records contain information relevant for outbreak investigations; consequently, by integrating the relevant data sources, we can potentially inform and improve patient screening and isolation strategies. However, this integration necessarily leads to large quantities of data, which can be difficult to analyze using standard statistical techniques. Machine-learning or “artificial intelligence” methods comprise a toolbox of approaches that have become popular for analyzing such “big data”.^1,2^ To date in the field of hospital epidemiology, machine-learning techniques have predominantly been used to extend existing statistical methods to provide deeper insights in the analysis of infections, infection management and outbreak detection (eg, Roth et al,^3^ Luz et al,^4^ and Leclère et al^5^). Furthermore, methods based on graph theory (hereafter “network graph methods”) have recently been used to identify, for example, superspreaders in community-based outbreaks.^6,7^

Here, we applied a network graph approach to the largest documented outbreak of a multidrug-resistant organism (MDRO) in Switzerland, which occurred in 2018–2019 in our hospital group and affected >560 patients.^8^ This particular MDRO, vancomycin-resistant *Enterococcus faecium* (VRE) of the sequence type 796 (ST796), predominantly colonizes the gastrointestinal tract, and is known for rapid intrahospital and interhospital spread. Infections due to VRE are associated with increased mortality, morbidity and higher hospital costs.^9,10^ Comprehensive literature has described the risk factors for VRE colonization including (among others) length of hospital stay, duration and type of antibiotic use, proximity to a colonized or infected patient, contact with environmental contamination, and immunosuppression or hematologic malignancy.^11–15^ The apparent complexity and multifactorial nature of this outbreak provided motivation for using machine-learning and graph-theoretical methods to attempt to untangle and better understand these complex interactions.

Active surveillance screening is a key measure in identifying asymptomatic VRE carriage, with patient contact isolation being the standard precaution to limit further transmission.^16–19^ Therefore, during this outbreak, VRE-positive patients (colonized or infected) were isolated, and a proactive “contact tracing” process was introduced. Although such contact tracing is relatively straightforward to implement, there was room for improvement and optimization of the process because transmission is not necessarily dependent only on rather limited definitions of geographical and organizational proximity.

With this study, we sought to address the following key questions:

- What are the risk factors for VRE colonization?
- Which patients should be screened?
- What are the “hot spots” in terms of devices, rooms and employees where transmission may have occurred?
- What is the potential benefit of a contact screening approach based on the network graph approach, compared to the traditional proximity-based contact screening?

To ease readability, throughout the document we have noted the use of standard statistical approaches versus new machine-learning–type techniques.

## Methods

The outbreak occurred in a 900-bed, tertiary-care, university hospital in Bern, Switzerland. The hospital sees ∼60,000 admissions and 380,000 patient days per year, with most medical disciplines represented.

The outbreak was originally detected in January 2018 following 2 cases of VRE bloodstream infections on the oncology ward, and this consequently led to an outbreak management protocol being introduced based on international guidelines. Briefly, VRE–positive (ie, colonized or infected) patients were isolated, and a proactive proximity-based contact-tracing process was introduced, whereby people were screened if they were hospitalized in the same room and ward (and therefore potentially exposed) as a newly detected VRE-positive patient in the prior 7 days. In addition, cleaning was intensified with measures such as daily disinfection and UV light cleaning procedures (among others). An upcoming publication describes the outbreak and procedures in more detail.^20^

In addition, we assembled diverse data from electronic medical records generated during the first 2 years of the outbreak (January 1, 2018–December 31, 2019), covering different aspects of medical care (Table 1). For comparisons, we labeled all patients (including children) with VRE acquisition during the outbreak period as VRE-positive (cases), and all other patients, whether tested or not, were assumed to be VRE negative (controls). Notably, a sampling and subtyping performed in 2018 revealed that 91.7% of the isolates were identified as sequence type 796; the analysis presented here pertains to all subtypes.

**Table 1.** Characteristics of Those With (Case) and Without (Control) VRE Infection (N = 87,244)

Patient Characteristics	VRE Positive (N = 560)	VRE Negative or Not Tested (N = 86,684)	*P* Value
Sex, male, no. (%)	346 (61.8)	44,019 (50.8)	<.001
Age, median y [IQR]	73 [63–82]	58 [33–75]	<.001
Length of stay (days), median [IQR]	8 [4–17]	4 [2–13]	<.001
ICU stay at any time, no. (%)	193 (34.5)	6,894 (8.0)	<.001
Mean Charlson score, median [IQR]	3 [2–5]	0 [0–2]	<.001
Patient had surgery at any time, no. (%)	377 (67.3)	49,581 (57.2)	<.001
No. of surgeries (all stays), median [IQR]	2 [1–4]	1 [0–1]	<.001
No. of different antibiotics taken during stay(s), median [IQR]	4 [2–6]	0 [0–1]	<.001
No. of rooms patient stayed in, median [IQR]	7 [4–11]	2 [1–4]	<.001
No. of hospitalizations, median [IQR]	3 [2–6]	1 [1–2]	<.001
**No. of contacts with different employees, no. (%)^a^**			<.001
None recorded	36 (6.4)	16,768 (19.3)	
1–5	131 (23.4)	36,465 (42.1)	
>5	393 (70.2)	33,451 (38.6)	
**No. of different medical devices encountered during stay, no. (%)**			<.001
None recorded	119 (21.2)	36,487 (42.1)	
1–5	270 (48.2)	42,209 (48.7)	
>5	171 (30.5)	7,988 (9.2)	

a “Employee contacts” means recorded interactions with nursing employees only.

Summary statistics of cases and controls were presented as number and percentage for categorical, and median and interquartile ranges for continuous, variables. Group differences were investigated using the χ^2^ test (or variants thereof) for categorical variables and the Mann-Whitney-Wilcoxon test for continuous variables.

### Standard approach

We identified risk factors for VRE colonization by fitting uni- and multivariable logistic regression models with dependent variable colonization (0 = no, 1 = yes), and independent variables those available and integrated in the project specific data warehouse (ie, those in Table 1). The most parsimonious model was found by forward selection then backward deletion using a *P* value of >.10 as the inclusion criterion. These analyses were performed in R version 3.5 or newer software^21^ using base functions and the following packages: *data.table*, *ggplot2*, *tableone*, and *survey*.

### Decision tree approach

In a second step, a machine-learning “decision tree” approach was used to identify a VRE positive individual. In decision-tree learning, a tree-like model of decisions and subsequent outcomes is chosen, which models the path from observations to conclusions. The branches of the tree represent criteria on the observations (eg, age <30 years), and the leaves represent the class labels (eg, those colonized or infected). The learning algorithm builds such a tree by choosing variables, and it learns criteria on these to split the data set most appropriately to the dependent variable, thus revealing the variables that are most predictive, as well as the tree that generates the predicted result. For our purposes, the classification tree was built with the same independent and dependent variables as the logistic regression (ie, those in Table 1).

This approach served to validate initial hypotheses of risk factors derived from the logistic regression models and provided an interpretable decision tree with decision thresholds. To mitigate the strong imbalance between cases and controls in the data set, we applied a minority class oversampling approach resulting in equal numbers of positive and negative samples. The analysis was performed using Python version 3.7 software^22^ with the following packages: data processing (*pyodbc*, *numpy*, *pandas*, *scipy*), machine learning (*scikit-learn*), and visualization (*matplotlib*).

### Network graph approach

In a third step, a network graph model involving the identified risk factors was developed. We identified potential transmission hotspots: healthcare employees with frequent VRE patient contact, rooms in which VRE patients were present, and medical devices used in conjunction with diagnosing and treating these patients. Interactions documented by healthcare workers were limited to those between patients and nursing employees because physician interactions are not documented at this granularity for in-patient care (which is a limitation of the data available to us). In terms of medical devices, only interactions tracked in the information technology systems were included in the analysis, and this limited the scope of this study to larger, often nonportable devices. Rooms in which patients stayed at any point during their hospitalization were included in the analysis, even if the stay was <24 hours.

The network graph analysis used eigenvector centrality to generate a daily “hotspot list”, which was sent to the relevant organizational unit involved in disinfecting rooms and medical devices. Employees remained as important links in the analysis, but hardly any employees underwent VRE screening by occupational health as it was deemed voluntary at the time.

**Fig. 1.**
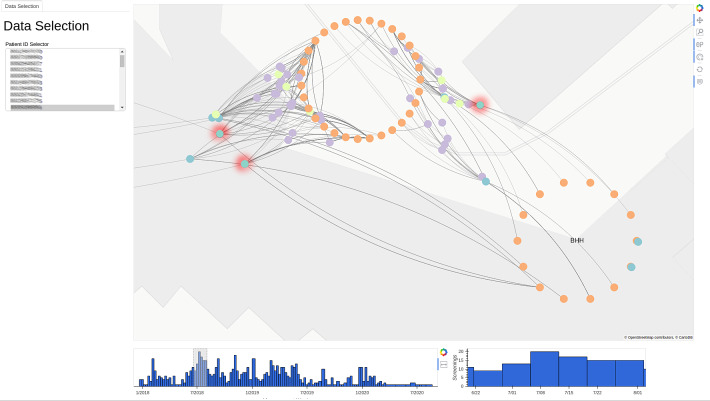
Example visualization shows collections of rooms in the geospatial locations in orange, patients in turquoise (colonized patients with red halo), devices in yellow, and employees in purple. In the left panel, it is possible to select a subset of all patients. In the bottom row, the user can select a subset of the timeline of VRE screenings.

An example of a small section of the network graph is shown in Figure 1. For background information on this approach, please refer to the Supplementary Material (online).

This analysis was performed using Python version 3.7 software^22^ with the following packages: data processing (*pyodbc*, *numpy*, *pandas*, *scipy*), graph theory (*networkx*), machine learning (*scikit-learn*, *pytorch*), and interactive visualization (*matplotlib*, *seaborn*, *bokeh*, *Holoviz*, *Holoviews*).

### Model validation

The outbreak occurred in many parts of the hospital, and the analysis was performed on data from the whole hospital. However, for the model validation process, we focused on the oncology ward because this ward performed screenings of all patients every week, whereas other wards did not or performed them only sporadically. This meant that for the oncology ward, we had a “ground truth” with which to compare our model predictions; that is, for each week we were able to compare the predicted VRE positive cases from the model to the observed cases.

We compared the current gold standard “proximity-based screening” and therefore nonprioritized approach, with the new prioritized list for screening patients derived from the network graph method. First, the “screening efficiency” was calculated; this represents the percentage fewer screenings compared to the gold standard. Second, the “screening benefit” was determined as the additional patients we could potentially have detected compared to the gold standard (refer to the Supplementary Material online for details).

### Ethical approval

The Cantonal Ethics Committee (Bern, Project ID no. 2020-00173) approved this study.

## Results

We compared 560 VRE patients to 86,684 controls from January 1, 2018, to December 31, 2019 (Table 1). Compared to the general hospital population during this period, VRE-positive patients were predominantly male (61.8% vs 50.8%), had an older median age (73 vs 58 years), had surgery during their hospitalization (67.3% vs 57.2%), and were more likely to have been in the ICU during their hospitalization (34.5% vs 8.0%).

### Standard approach

**Fig. 2.**
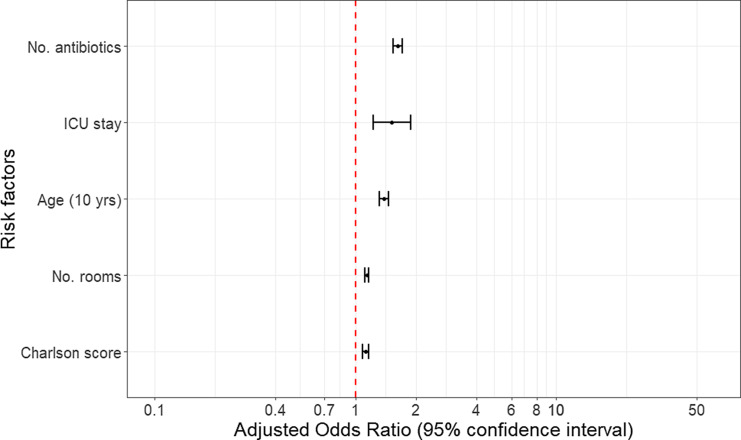
Forest plot of risk factors for VRE acquisition from the adjusted multivariable logistic regression model.

Independent predictors of VRE colonization from the fitted multivariable logistic regression model were age (adjusted odds ratio [aOR], 1.4 (per 10 years); 95% confidence interval [CI], 1.3–1.5; *P* < .001), ICU admission during any hospitalization (aOR, 1.5; 95% CI, 1.2–1.9; *P* < .001), Charlson comorbidity score (aOR, 1.1; 95% CI, 1.1–1.2; *P* < .001), number of different prescribed antibiotics (aOR, 1.6; 95% CI, 1.5–1.7; *P* < .001) and the number of rooms the patient stayed in during the study period (aOR, 1.1; 95% CI, 1.1–1.2; *P* < .001), which is a marker for potential multiple exposures to environments and also severity of illness (Table 2 and Fig. 2). Number of hospitalizations, number of employee contacts, and number of devices employed for care were also significant predictors in univariable models, but these were collinear with the “ICU” indicator and “number of rooms” variables in multivariable adjusted models.

**Table 2.** Estimated Risk Factors for VRE Infection From the Fitted Logistic Regression Model

Characteristic	Univariable		Multivariable	
No. of patients	Estimate (95% CI)	*P* Value	Estimate (95% CI)	*P* Value
**Sex**				
Female	1 (Ref)			
Male	1.6 (1.3–1.9)	<.001	…	NS
Age (10 y)	1.4 (1.4–1.5)	<.001	1.4 (1.3–1.5)	<.001
Length of stay (per 5 d)	1.0 (1.0–1.0)	.9	…	…
ICU stay (at any time)	6.1 (5.1–7.2)	<.001	1.5 (1.2–1.9)	<.001
Charlson score (mean)	1.4 (1.3–1.4)	<.001	1.1 (1.1–1.2)	<.001
Patient had surgery at any time	1.5 (1.3–1.8)	<.001	NE	…
No. of surgeries, all stays (per surgery)	1.2 (1.2–1.2)	<.001	…	…
No. of different antibiotics	2.1 (2.1–2.2)	<.001	1.6 (1.5–1.7)	<.001
No. of rooms (per room)	1.3 (1.3–1.4)	<.001	1.1 (1.1–1.2)	<.001
No. of hospitalizations	1.3 (1.3–1.3)	<.001	…	NE
**No. of contacts with different employees** None 1–5 >5	1 (Ref) 1.7 (1.2–2.5) 5.5 (4.0–7.8)	.006 <.001	…	NE
**No. of different medical devices encountered during stay** None 1–5 >5	1 (Ref) 2.0 (1.6–2.4) 6.6 (5.2–8.3)	<.001 <.001	…	NE

Note. Ref, reference; NS, not significant at the 5% level; NE, not estimated since the variable is collinear (no. of surgeries with the ICU indicator, no. of rooms with hospitalizations, no. of employees and devices with no. of rooms).

### Decision tree approach

Many of the risk factors identified in the logistic regression analysis are already well established in the literature. However, the presence of complex interactions between variables exhibited by the multiple collinearities in the fitted statistical models motivated a more in-depth investigation using machine learning methods. Decision-tree–based machine-learning methods (with a maximum depth of 6 levels) confirmed the findings from the logistic regression modelling, with the number of antibiotics (importance, 0.21), Charlson score (importance, 0.19), and number of rooms stayed in (importance, 0.15) being the 3 most important patient-associated characteristics (Table 3). The “importance” is a metric defining the rank of this covariate in splitting the data set: the higher the importance, the earlier this covariate is used in the branching process. As an additional result, the criteria of the decision tree provide proper thresholds that split the data appropriately. Furthermore, the analysis allowed insights that were not apparent from the logistic regression; for example, those patients receiving antibiotics and those aged >55 years were at higher risk for colonization (Supplementary Fig. S3 online).

**Table 3.** Estimated Features From Decision-Tree Analysis

Characteristic	Importance
No. of different antibiotics	0.214
Charlson score, mean	0.191
No. of rooms	0.145
Age	0.107
Charlson score (last)	0.104
No. of hospitalizations	0.067
No. of surgeries, all stays (per surgery)	0.066
Length of stay (per 5 days)	0.064
ICU stay (at any time)	0.020
Sex	0.011
Patient had surgery at any time	0.010

Note. ICU, intensive care unit.

### Network graph approach

The subsequent complex network analysis established 3 possible pathways by which the 560 VRE cases are connected (although not necessarily in a causal relationship): healthcare personnel, medical devices, and patient rooms. A sample “hot spot list” for a particular day is shown in Table 4; this ordered list of medical devices, healthcare personnel, and patient rooms has been sorted in the order of the likeliness of being colonized with VRE. Depending on the situational environment, appropriate actions can be defined regarding how to isolate patients and clean the devices and rooms more frequently and/or thoroughly, or potentially also to screen the respective personnel (although the latter was not implemented in this study).

**Table 4.** Example Hotspot List of Rooms, Devices, and Employees^a^

Node Identification	Node Type	Score
EKG service	Room	9.5
Examination room ZZ	Room	8.0
14252	Device	6.9
Operating room ZY	Room	3.5
BX04	Room	2.3
F123	Room	2.2
A	Employee	2.1
Operating room Y	Room	2.1
B	Employee	2.0
Gastro1	Room	2.0
ZHS-01	Room	1.7

Note. EKG, electrocardiogram.

a This shows that the electrocardiogram service and the examination room ZZ have many interactions, and thus could likely serve as carriers for transmission.

### Screening efficiency and benefit

In terms of model validation, the screening efficiency was estimated to result in 40.0% fewer screenings compared to the current gold standard (95% CI, 17.3–54.6). With the new screening approach, we identified 102 (95% CI, 66–138) positive patients who were missed from the proximity based screening process and who had an equal or higher centrality as the lowest-ranked positive patient.

## Discussion

We originally set out to address a number of key questions with respect to the outbreak and its investigation. We now address each of these in turn.

### What are the risk factors for VRE colonization?

Using both standard statistical methods and machine learning, we identified risk factors for VRE colonization in line with those already published in the literature. Patient age, underlying diseases and severity of illness, prolonged hospitalization, surgery, and exposure to antimicrobial drugs were important factors.^23–25^ Using 2 approaches confirming essentially the same results might be considered an inefficient use of statistical resources, but we prefer to view the 2 approaches as complementary, providing a slightly different perspective to the analysis. Although this was ultimately not particularly beneficial here, this will not always be the case.

### Which patients should be screened?

Using a complex network graph analysis, we were able to further investigate 3 main pathways by which the VRE cases are connected: healthcare personnel, medical devices, and patient rooms. Interestingly, the importance of the number of rooms a patient stayed in and patient device interactions reflects recent work by Weterings et al^26^ and Gouliouris et al,^27^ which identified environmental contamination (and cleaning) as important factors in VRE outbreaks.

Using a network-graph approach to identify super-spreaders is not a new concept^28,29^; to date, however, there have been few applications using medical data, and, to our knowledge, these approaches have not been applied to nosocomial outbreaks. Our centrality-based screening is theoretically related to that of Klemm et al,^30^ which suggests that the dynamic influence of a node in the classical susceptible–infected–recovered (SIR) transmission model can be estimated using eigenvector centrality.

### What are the “hot spots” in terms of devices, rooms and employees where transmission may have occurred?

The analysis identified probable “hot spots” based on the hospital system’s human and nonhuman connections. This yielded prioritized lists of rooms and devices that might require special measures for outbreak control.

### What is the potential benefit of a contact screening approach based on the network-graph approach, compared to the traditional proximity-based contact screening?

We proposed potential approaches for estimating the screening efficiency and the benefits of a more focused screening approach. These could certainly be improved, and we would recommend investing adequate time in defining appropriate metrics at the project initiation stage.

In more general terms, the network-graph analysis builds on the results from the logistic regression and decision-tree methods by identifying specific patients to be screened and rooms and devices to be cleaned.

Although the network-graph method was generally successful, the lack of a fully integrated data warehouse was a considerable barrier to the speed of implementation. For example, the time and resource management system did not include information on physicians and other key personnel within the hospital, so “employee” interactions were limited to those performed by nursing healthcare professionals. This represents an example of the main (financial and otherwise) cost of implementing such an approach: an important prerequisite for fast implementation (and therefore actionable results) is the availability of a hospital-wide, fully integrated, data warehouse (refer to the “blueprint” in the Appendix online). The most time-consuming and resource-intensive part of the project was the data source integration (Appendix, step 2) and operationalization (step 7), the necessary effort for which surprised the research team (Supplementary Fig. S2 online). The idea to pursue “machine-learning” methods is currently popular, but this can often lead to an analysis-driven approach. With this in mind, and given that, at least anecdotally, “85% of data science projects fail,”^31^ one of the most important success factors was to have a clear project mandate with metrics for success prior to starting the project.

This study had several limitations. Our study was monocentric, and it could have been improved by validating against data from another hospital. Despite the limitations, even our incomplete picture provides additional valuable insights regarding specific known interactions with VRE-positive patients in the greater network displayed in our research.

In summary, we implemented a new approach to reduce unnecessary screening. The method provides the basis for a smart contact-tracing system for the next outbreak, independent of pathogen, and potentially also for data-driven outbreak monitoring. We calculated the benefits of the new method compared to the existing contact tracing. The differential can be understood as an “added value” in terms of the yield of screening, or analogously, the cost–benefit of “avoided” screening.

Finally, data science provides a better understanding of outbreaks, but interpretations should include consideration of data source maturity, the scope of included sources, and potential confounding factors.

## Supplementary material

For supplementary material accompanying this paper visit https://doi.org/10.1017/ice.2022.66. click here to view supplementary material
