# Supplementary material for: Extending outbreak investigation with machine learning and graph theory: Benefits of new tools with application to a nosocomial outbreak of a multidrug-resistant organism
Source: Infect Control Hosp Epidemiol. 2022 Sep 16;44(2):246–52. doi: 10.1017/ice.2022.66 (PMC9929710; doi:10.1017/ice.2022.66)

Supplementary material

Appendix: Network graphs

Network graphs are omnipresent data structures, which underlie many problems of computer science, but also extend to research questions in medicine, biology and social sciences. Examples such as protein networks, knowledge-relational graphs or social networks, but also, as presented here, interactions in a hospital can be modelled as graphs. Each unit (e.g. an infected patient) is represented as a “node” in a graph, and interactions with other entities (e.g. rooms, employees or medical devices) are shown as “edges” in the graph. To understand such a network’s properties, network graph methods identify, for example, influential people, key resources or disease super-spreaders. The underlying principles of these phenomena are hidden in the connectivity among the nodes, and can be revealed through so-called “network centrality metrics”. In general, these address the key question of determining the characteristics of an important node, and thus incorporate different properties of node connectivity. Classical centrality metrics are the degree centrality (i.e. number of incoming/outgoing edges), betweenness centrality (i.e. number of random paths through a particular node), or eigenvector centrality (relative node importance based on number of incoming edges of other important nodes). They are applied to determine the central nodes for different problems, such as identifying popular people in a social network (degree centrality), determining critical infrastructure in a computer network (betweenness centrality), or finding relevant websites for a topic based on other relevant websites linking to it (eigenvector centrality). An illustrative example of these metrics is shown in Figure S1.

Figure S1: Centrality metrics explained

Panel A: Examples of the different centralities applied to a synthetic network: Betweenness centrality (top left); Closeness centrality (top right); Eigenvector centrality (bottom left); Degree centrality (bottom right), respectively. This plot shows how different centralities can determine strongly differing concepts of a node being “central”; blue having lowest centrality, red highest centrality, with a sliding scale of colours/centrality between.


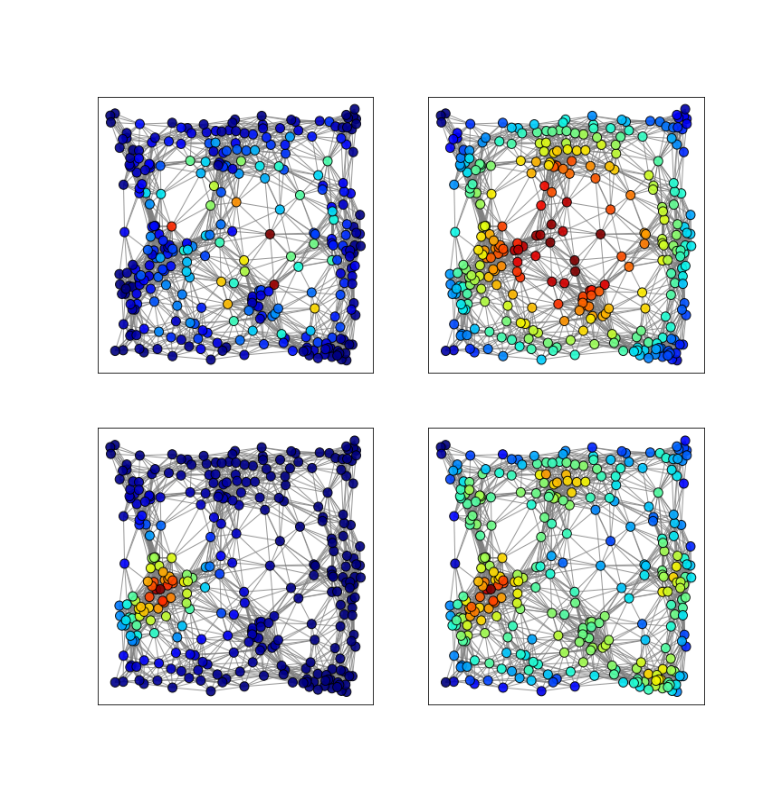


Panel B: Example of a small interaction graph and the intuition of the betweenness centrality for Room 1 for all paths between colonized patients (1, 2 and 3). The edges in purple show the shortest paths between these patients that all go through Room 1. Thus, Room 1 could constitute a carrier for VRE transmission. The betweenness centrality identifies such transmission routes, and builds a proxy for transmission importance. Nodes with high betweenness centrality should undergo more frequent decontamination measures.

Appendix: Blueprint for outbreak investigation

An inter-disciplinary team of people with expertise in data architecture and transformation, IT project management, data science, biostatistics, infectious disease modelling, and clinical skills, was assembled. The following is a step-by-step definition of the process we followed:

- Step 1:
  - Definition of endpoints and deliverables
  - Definition of what constitutes “success” for the project.
  - Definition of which data were relevant and should be included in the project specific data warehouse.
- Step 2: Data collection (from existing source systems and applications)
- Step 3: Data cleaning, integration and aggregation in the data warehouse, with particular focus on facilitating operational processes; downloads from source databases required at least once daily; requiring a secure, robust platform with interfaces to source systems; change control in upstream databases is recorded and passed downstream to the data warehouse.
- Step 4: Analysis
  - Statistical analysis: What are the main risk factors for infection? (e.g. logistic regression analysis)
  - Machine Learning: Refine statistical analysis providing additional insights (e.g. decision trees).
  - Network Graph: What are the main transmission pathways for infection? (e.g. network graph theory).
- Step 5: Visualisation of the transmission network. This is of vital importance to bridge the gap between those with clinical/epidemiological expertise, and those implementing the methods; also helps in acquiring buy-in from key management stakeholders funding the project.
- Step 6: Predictive modelling including verification and validation process
- Step 7: Operationalization
  - Provision of IT infrastructure (data warehouse incl. Hadoop cluster, SQL Server (MSSQL), Docker operationalization platform, Airflow scheduler, Python with Data Processing (pyodbc, numpy, pandas, scipy), Graph Theory (networkx), ML (scikit-learn, pytorch) and interactive Visualization (matplotlib, seaborn, bokeh, Holoviz, Holoviews), SQL Server Reporting Service (SSRS)).
  - Integration of hotspot lists into existing process chain
  - Definition of exclusion list of spurious rooms/medical devices.
  - Production of hotspot lists
  - Actions items based on the hotspot list
- Step 8: Benchmarking and cost-benefit analysis
  - What is the difference between the traditional contact tracing and network tracing based approach?
  - What is the additional benefits of using network graph analysis in terms of patient screening: which patients did we find through network analysis that we did not find through contact tracing? This is vitally important to the success of the project.

Figure S2: Estimated resource allocation in percentage of total time used in project

Figure S3: Excerpt from the decision tree with branching point for “age over 55” (left hand side, bottom) followed by taking antibiotics.


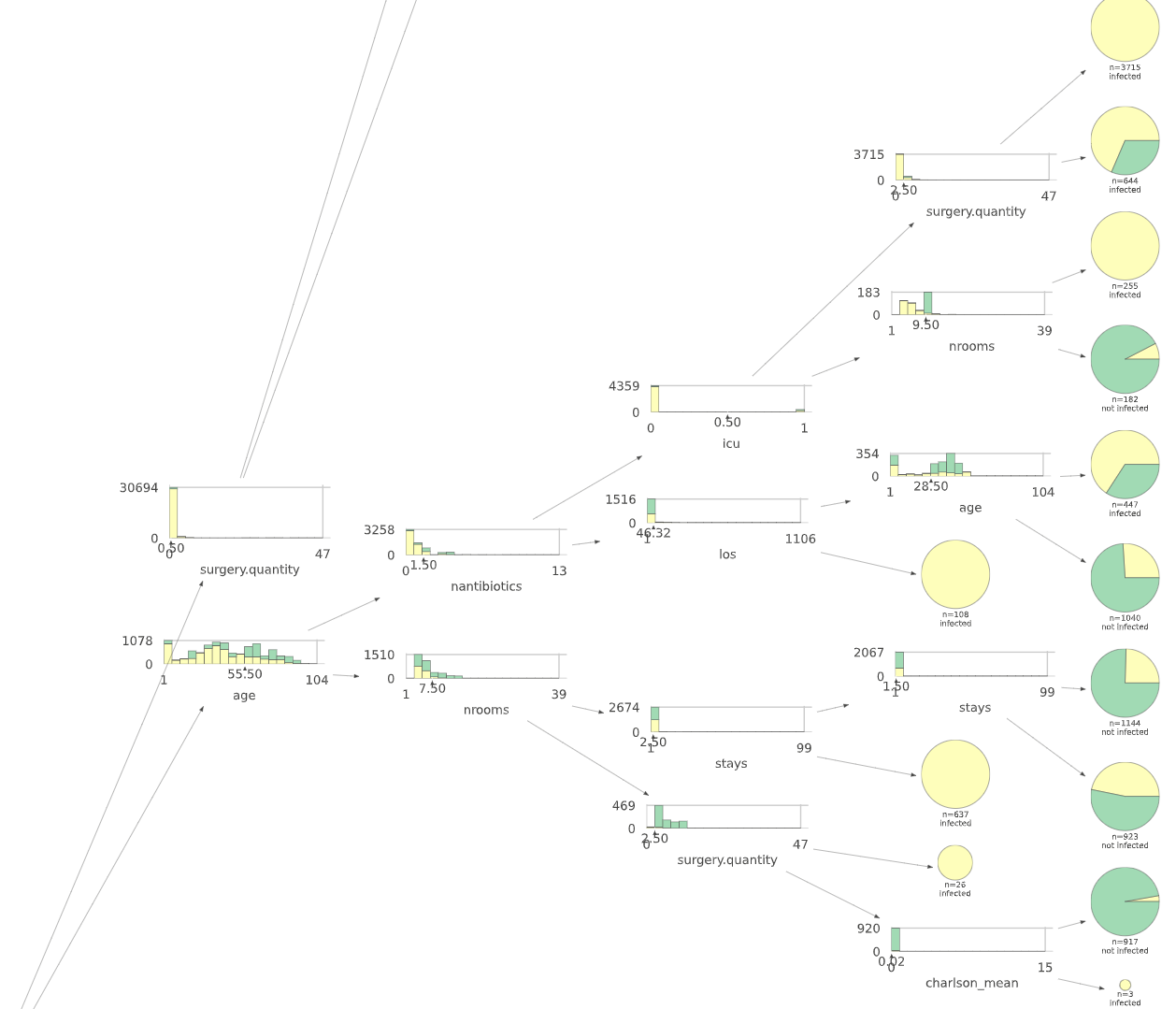

Supplement: Supplementary file 1 [file S0899823X22000666sup001.docx]
